# Apple Fruit Edge Detection Model Using a Rough Set and Convolutional Neural Network

**DOI:** 10.3390/s24072283

**Published:** 2024-04-03

**Authors:** Junqing Li, Ruiyi Han, Fangyi Li, Guoao Dong, Yu Ma, Wei Yang, Guanghui Qi, Liang Zhang

**Affiliations:** 1 College of Information Science and Engineering, Shandong Agricultural University, Tai’an 271018, China; junqing.li@sdau.edu.cn (J.L.);; 2 College of Computer Science and Technology, China University of Petroleum (East China), Changjiang Road No.66, Qingdao 266580, China; 3 College of Information and Electrical Engineering, China Agricultural University, Beijing 100083, China; 4 National Key Laboratory of Wheat Improvement, College of Life Science, Shandong Agricultural University, Tai’an 271018, China

**Keywords:** rough set, Faster-RCNN, edge detection, target detection, apple fruit

## Abstract

Accurately and effectively detecting the growth position and contour size of apple fruits is crucial for achieving intelligent picking and yield predictions. Thus, an effective fruit edge detection algorithm is necessary. In this study, a fusion edge detection model (RED) based on a convolutional neural network and rough sets was proposed. The Faster-RCNN was used to segment multiple apple images into a single apple image for edge detection, greatly reducing the surrounding noise of the target. Moreover, the K-means clustering algorithm was used to segment the target of a single apple image for further noise reduction. Considering the influence of illumination, complex backgrounds and dense occlusions, rough set was applied to obtain the edge image of the target for the upper and lower approximation images, and the results were compared with those of relevant algorithms in this field. The experimental results showed that the RED model in this paper had high accuracy and robustness, and its detection accuracy and stability were significantly improved compared to those of traditional operators, especially under the influence of illumination and complex backgrounds. The RED model is expected to provide a promising basis for intelligent fruit picking and yield prediction.

## 1. Introduction

The apple is one of the most economically important perennial crops cultivated worldwide. China is the largest apple growing and consuming country, accounting for 41.36% and 46.85% of world’s apple planting area and production, respectively [1]. The development of the apple planting industry constantly proposed higher requirements for apple fruit growth detection and intelligent picking, and accurate identification and detection of apple fruits are among the key technologies urgently needed.

Improving the target location and edge detection accuracy of apple fruit in images is one of the key and most difficult points in the current research. Thus, a number of researchers have performed a lot of work on this topic. In this section, we summarized the application of traditional morphological edge detection methods and artificial intelligence methods in apple edge detection, as well as the efforts made in apple fruit target detection and segmentation tasks.

In terms of traditional morphological edge detection methods, Versaci et al. proposed a new edge detection algorithm based on fuzzy divergence and fuzzy entropy minimization, which can effectively solve the problem of edge detection in fuzzy images [2]. Han et al. proposed an improved edge detection algorithm based on Sobel operator, which can effectively solve the problems of low image edge localization accuracy and rough edge extraction in traditional Sobel edge detection algorithms [3]. Lu et al. proposed an edge detection algorithm based on the Canny operator, which introduced a local maximum inter class variance algorithm to achieve efficient edge detection of infrared images of building exterior walls [4]. Sekehravani et al. proposed an edge detection algorithm combining median filtering and Canny operator, which can improve the accuracy of edge detection in noisy images [5]. Septiarini et al. proposed a contour-based segmentation method for oil palm fruits that removes noise through multiple operations by combining the Canny algorithm, morphology, and reconstruction. However, in images containing multicolored oil palm fruits, complex backgrounds, and uneven lighting conditions, there is a problem of incorrectly detecting fruit regions as backgrounds [6]. Jiao et al. conducted research on issues related to apple growth poses in natural scenes, such as overlapping, target shadow occlusion, and leaf occlusion. They proposed an overlapped circle positioning method based on local maxima for fruit localization and edge delineation. However, errors in the center positioning process can lead to deviations in the final edge delineation, especially in cases of fruit occlusion and influence from surrounding non-target factors, exacerbating the deviation [7].

In terms of fruit edge detection with artificial intelligence methods, Su et al. proposed a simple and lightweight network structure called the Pixel Differential Network (PiDiNet) by integrating traditional edge detection operators into popular convolutional operations in modern CNNs, which can quickly and accurately perform edge detection while minimizing the number of parameters as much as possible [8]. Wang et al. used a natural statistical visual attention model to remove the background and combined the information with the global probability of the Otsu algorithm to detect saliency contours, which addressed the problems of uneven and mutual occlusion of apple images in orchard environments [9]. Ganesan et al. proposed a method that combines mountain climbing and the MFCM to segment fruits in RGB and CLELuv color spaces; this method solves the problem of the mountain climbing method falling into local optima [10]. Xavier Soria et al. proposed an edge detector based on deep learning, which can be used for any edge detection task by combining holistically nested edge detection (HED) and Xception networks without pre-training or fine-tuning processes [11].

In the related work of apple target detection and segmentation, Wang et al. proposed an improved convolutional neural network (MS-ADS), based on masked scoring regions, for precise apple detection and instance segmentation in natural environments. However, the improvement in accuracy brought by this work also leads to an increase in the model’s complexity, resulting in longer training and detection times. Moreover, it requires a larger dataset to encompass real-world scenarios [12]. Tian et al. improved the YOLO-V3 model to effectively perform apple fruit object detection tasks across different growth stages in natural scenes. The experimental results indicated that the model exhibited improved performance; however, there is still room for improvement in data augmentation techniques and detection model accuracy [13]. Li et al. proposed an ensemble U-Net segmentation model suitable for small-sample datasets. This model integrates residual blocks, gated convolution for edge structure design, and employs atrous convolution to achieve superior fruit segmentation results. However, overcoming branch and leaf occlusion, as well as improving model speed, still require further enhancements [14]. Zhang et al. proposed an apple fruit segmentation method based on machine learning techniques. Unlike deep learning methods, this approach relies on color features and texture information such as the gray-level co-occurrence matrix (GLCM). Through traditional machine learning methods like random forest, it achieves a precision rate of 94%. This machine learning-based method outperforms CNN methods in terms of training and time efficiency. However, it is slightly inferior to CNN methods in terms of precision and overcoming environmental noise [15].

The rough set theory is a mathematical tool for addressing vague and uncertain problems, and its characteristics are highly suitable for edge detection. However, there is still much room for improvement in the application of rough set theory in edge detection.

The current fruit edge detection algorithms are not very effective at dealing with noise caused by natural factors such as illumination and occlusion and have poor stability in processing complex background images. Therefore, a reasonable background removal model was proposed in this paper that combined the characteristics of rough set to detect apple fruit edges effectively.

In the natural environment, the target recognition process is strongly affected by the interference background of illumination, sky, branches, and leaves, as well as the mutual occlusion of apples themselves, which greatly increases the difficulty in locating targets and directly affects fruit edge recognition and detection accuracy. In this paper, a fruit edge detection model based on the fusion of a rough set and the Faster-RCNN was proposed. The main contributions of this work are as follows:

(1) The object detection algorithm based on the Faster-RCNN was applied to denoise complex environments around fruits, enhancing its robustness to noise such as sky background and illumination.
(2) Clustering and morphological methods were used to supplement the voids and incomplete information caused by branch and leaf obstructions in the images.
(3) Rough set was introduced to extract complete and continuous fruit edges by utilizing the edge information contained in the upper and lower approximations of the image.

## 2. Materials and Methods

### 2.1. Data Acquisition and Preprocessing

**Figure 1:**
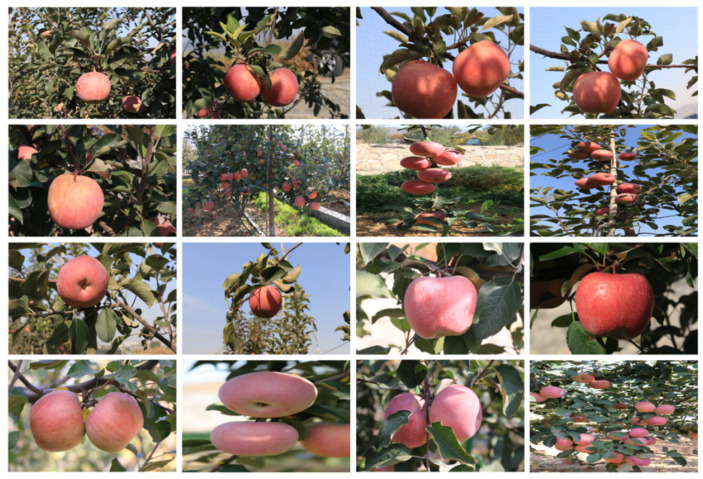
Partial images in the dataset.

The entire dataset included 1500 images obtained via natural environment photography and online image collection. Part of the dataset is shown in Figure 1. In this study, 1200 images were selected as the training set, and 300 images were used as the testing set. In the preprocessing stage, the label learning tool was used to annotate the dataset.

**Figure 2:**
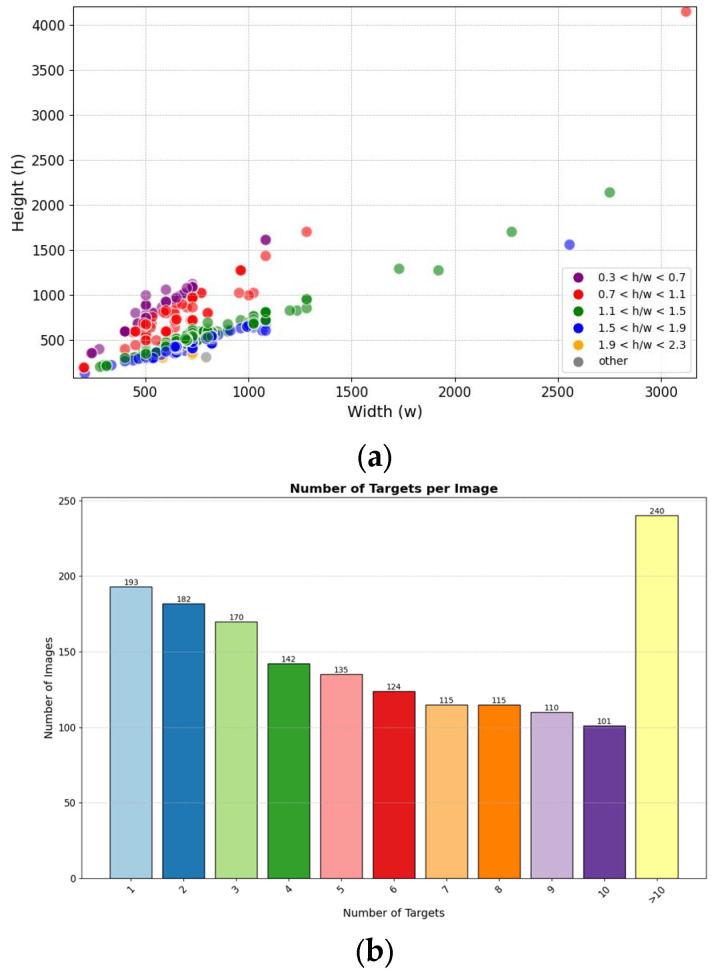
Dataset description. (**a**) shows the resolution statistics and distribution of images in the dataset. (**b**) shows the statistical analysis of the number of targets contained within each image in the dataset.

We conducted further analysis on the content of the dataset. The statistical analysis and distribution of image resolutions in the dataset is shown in Figure 2a. The distribution of the number of targets contained within each image in the dataset is shown in Figure 2b.

**Figure 3:**
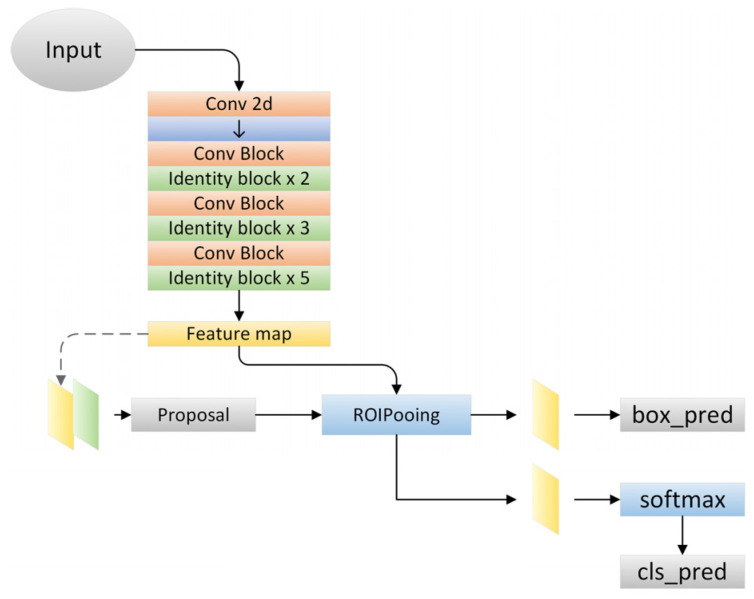
Faster-RCNN model architecture diagram.

In this study, the Faster R-CNN model was constructed based on Keras and TensorFlow, as shown in Figure 3. We utilized Res2Net-50 as the backbone for training, and conducted training for 100 epochs. During the training process, we employed the Adam optimizer with a batch size of 16. The input image size was set to 800 × 1333, and we applied data augmentation techniques such as random rotation (−15°~15°), random flipping, and jitter, etc., during the training process. The computer processor was an Intel (R) core i7-H8750 (Intel, Santa Clara, CA, USA), with a memory of 16.00 G and a frequency of 2.20 GHz.

**Figure 4:**
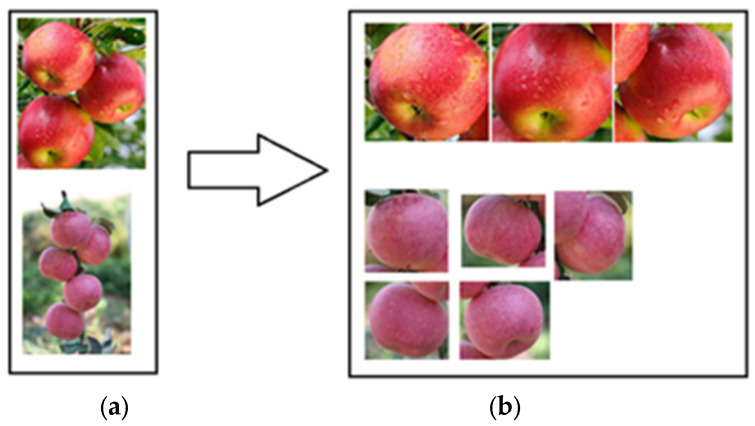
Display diagram of apple target detection model recognition segmentation. (**a**) shows the original input image and (**b**) shows multiple apple images for testing image segmentation.

In this study, a manually annotated apple image dataset was used to train the model, and complex apple images obtained from natural environments were used as the test set for accuracy testing. The results of the apple object detection are shown in Figure 4.

### 2.2. Background Knowledge

#### 2.2.1. Faster-RCNN

Faster-RCNN is a target detection algorithm presented by He Kaiming et al. [16], and many researchers have improved it [17,18,19]. The algorithm is divided into four main parts: a feature extraction network, a PNN, a pooling layer, and a classification layer.

The backbone feature extraction networks commonly used in the Faster-RCNN network architecture include VGG [20] and ResNet [21]. ResNet has a larger network than VGG, so it has a strong learning ability and significantly improved classification. ResNet 50 was used as the training backbone feature extraction network in this paper.

The region proposal network (RPN) [22] is an advantage of Faster-RCNN, which greatly improves the generation speed of detection frames. The method includes three steps: obtaining positive and negative classifications through SoftMax classification anchors; obtaining accurate proposals by calculating the bounding box regression offset of anchors; obtaining proposals by combining the first two steps, and removing proposals that are too small or beyond the boundary.

The classification section is the classification layer of the Faster-RCNN, which can be used to identify multiple targets and classify them. Given that this paper mainly focused on apple localization and recognition, the training category was the apple class.

#### 2.2.2. Image Color Space Conversion

The color space [23] is the theoretical basis for color information research. This space quantifies color from people’s subjective feelings into specific expressions, providing a strong basis for using computers to record and express color.

The conversion from the RGB color space to the LAB space usually starts with the transition to the XYZ color space and then further converts to the LAB space. The conversion formula is as follows [24].

RGB color space to the XYZ color space:

(1) $$\left\{\begin{array}{c} X=0.412453R+0.35780G+0.180423B\;\;\;\;\;\;\;\;\;\;\;\\ \;Y=0.212671R+0.715160G+0.072169B\;\;\;\;\;\;\;\;\;\;\\ Z=0.019334R+0.119193G+0.950227B\;\;\;\;\;\;\;\;\;\; \end{array}\right.$$

XYZ color space to LAB color space:

(2) $$\left\{\begin{array}{c} L=116f\left(\frac{Y}{Y_{n}}\right)-16\;\;\;\;\;\;\;\;\;\;\;\;\;\;\\ a=500(f\left(\frac{X}{X_{n}}\right)-f\left(\frac{Y}{Y_{n}}\right))\\ b=200(f\left(\frac{Y}{Y_{n}}\right)-f\left(\frac{Z}{Z_{n}}\right)) \end{array}\right.$$

where $X_{n}$, $Y_{n}$, and $Z_{n}$ are the CIE XYZ tristimulus from the white point reference, each of which is $X_{n}$ = 0.950456, $Y_{n}$ = 1.0, and $Z_{n}$ = 1.088754.

#### 2.2.3. K-Means Clustering

The K-means clustering algorithm is an iterative clustering analysis algorithm that is suitable for unsupervised learning dataset analysis and has strong adaptability; thus, it is widely used in image segmentation [25,26,27].

Assume that the target image I contains N pixels, each pixel in I is represented as $X_{i}$ (1≤i≤N), and the three channel L, a, and b values contained in each pixel are the eigenvalues of $X_{i}$ respectively. The pixel set of the same image is denoted as $D=\left\{X_{i}|X_{i}\in I,1\leq i\leq N\right\}$.

#### 2.2.4. Dilation and Erosion of Images

Dilation and erosion are the most basic operations for determining morphology, and a reasonable combination of these operations can reduce the noise around the apple and minimize the loss of the target image.

Erosion is defined as $A⊙B=\left\{x|{\left(B\right)}_{x}\subseteq A\right\}$. For binary image A of the target after clustering, convolutional template B is used for erosion treatment. The convolutional calculation is performed between templates B and A to obtain the minimum pixel value in the coverage area of B in A, and this minimum value is used to replace the pixel value of the reference point.

Dilation is defined as $A⊕B=\left\{x|(B{)}_{x}\cap A\neq \emptyset \right\}$. For the target image C, a convolutional template D is used for dilation processing. A convolution calculation is performed between template D and image C. The AND operation is performed on each pixel in the scanned image. Scan each pixel in the image and perform an AND operation. If the results are all 0, the target pixel is 0. Finally, the maximum pixel value of the D coverage area in C is obtained, and this maximum value is used to replace the pixel value of the reference point.

#### 2.2.5. Rough Set

The rough set (RS) theory [28,29,30] is a mathematical tool for characterizing undefined and uncertain information. This theory can be used to analyze and process various incomplete information such as imprecision, contradiction, and incompleteness effectively and obtain implicit knowledge and rules [31]. RS theory was developed by the Polish scientist Z Pawlak in 1982. The related concepts and definitions of RS are as follows.

Discourse domain U: given a finite nonempty set, the classification knowledge is embedded in the set.

Knowledge: the ability to classify objects. The object here refers to any entity, namely, the discourse domain, which is any subset family of U.

Knowledge base: the classification family on U is called the knowledge base.

Knowledge equivalence: ind(P) = ind(Q), indicating that P is equivalent to Q. P and Q are two equivalence relation families defined on the set U.

**Definition** **1.** *For a given finite nonempty set U, R is an equivalent relationship in U, also known as R’s knowledge about U.*

For a rough set, whether an object x belongs to set X can be divided into the following situations: ① x definitely does not belong to X; ② x definitely belongs to X; ③ x may or may not belong to X. Based on the above three situations, use ${\left[x\right]}_{R}$ to represent the set of all objects that are indistinguishable from x. Provide definitions for the upper approximation, lower approximation, negative field, and boundary of set X.

(3) $$\left\{\begin{array}{c} L\left(X\right)=\left\{x\in U:{\left[X\right]}_{R}\subseteq X\right\}\;\;\;\;\;\;\;\;\;\;\;\;\;\;\\ U\left(X\right)=\left\{x\in U:{\left[X\right]}_{R}\cap X\neq \emptyset \right\}\;\;\;\;\;\\ Bn\left(X\right)=U\left(X\right)-L\left(X\right)\;\;\;\;\;\;\;\;\;\;\;\;\;\;\;\;\;\;\;\;\\ Neg\left(X\right)=\left\{x\in U:x\notin U\left(X\right)\right\}\;\;\;\;\;\;\; \end{array}\right.$$

**Figure 5:**
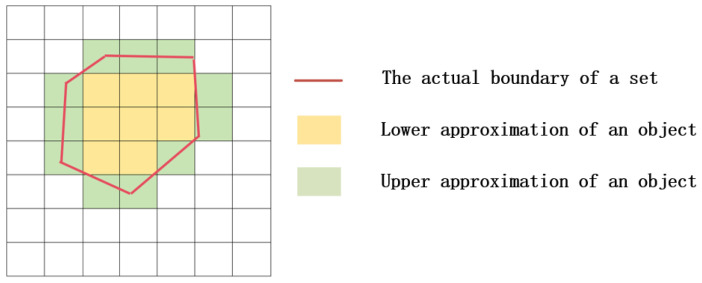
The upper and lower approximation graphs of rough set.

The upper and lower approximation graphs of rough set are shown in Figure 5. The curved section in Figure 1 represents the true boundary of the identified object. The internal area of this boundary is the lower approximation L (X) of the object, which represents the smallest definable set (positive field) that may exist in the physical position of the object. The green area that intersects with the true boundary is called the boundary domain BN (X) of the object. The area outside the boundary domain is the negative domain Neg (X) of the object, which means that the physical location of the object must not be within that area. Negating the set Neg (X) yields the upper approximation U (X) of the object, which represents the maximum definable set in which the physical position of the object may exist.

### 2.3. Overall Architecture of Our Edge Detection Model (RED)

**Figure 6:**
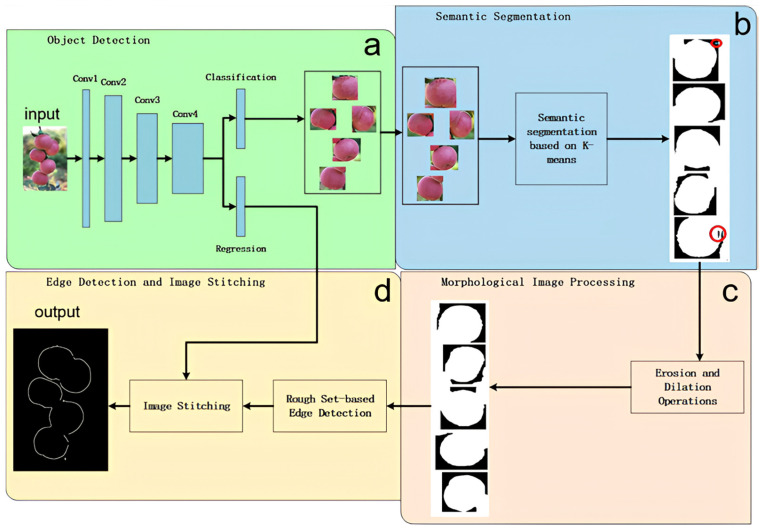
The basic architecture diagram of RED. The overall algorithm consists of four modules: (**a**) object detection module, (**b**) semantic segmentation module, the red circles marked in the segmented image (right subplot of b) indicate the holes and noise that need to be addressed after segmentation, which will be handled in module c, (**c**) morphological image processing module, and (**d**) edge detection and image stitching module. The initial input comprises multiple images of apples in natural environments, and the final output is the corresponding edge detection result image.

This model focuses on accurate detection of the edges of apple fruit targets. Therefore, in the process of image processing, attention should be given to removing as much noise as possible and minimizing the loss of target features. The basic architecture diagram of RED is shown in Figure 6.

First, the Faster-RCNN algorithm was used to detect the position of apples in natural environment apple tree images. Then, the RGB image was converted into a LAB color space, and the features of the apples were extracted through K-means clustering. Next, morphological methods such as corrosion, expansion, and void filling were used to process the edges and internal noise, and the edge image of a single apple was extracted using a rough set to obtain the upper and lower approximations of the edges. Finally, the edge images of all the individual apples were merged to form a complete edge image that includes all the apples.

### 2.4. Detection Module

**Figure 7:**
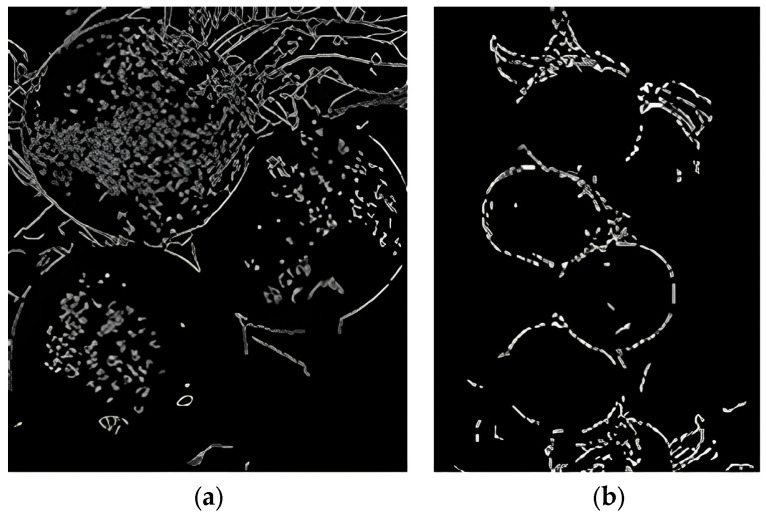
Effect of edge detection with a large amount of noise. Both (**a**) and (**b**) are the results of edge detection directly on the initial natural environment image, and the outcomes appear to be unsatisfactory.

Most of the images in the dataset contain multiple apples, as well as irrelevant features, such as sky background, branches, and leaves. Direct edge detection resulted in a large amount of noise in the results (as shown in Figure 7). In response to this issue, the Faster-RCNN was used to perform individual apple detection and segmentation operations on each image, removing irrelevant features and transforming the multi-apple edge detection problem into a single apple edge detection problem.

### 2.5. Refinement Module

The refinement module was the key part of this study. The output of Faster-RCNN was used as the input of the edge detection model, and significant targets were obtained through spatial transformation, clustering segmentation, and image morphology processing after segmentation.

#### 2.5.1. Apple Image Segmentation Based on K-Means Clustering

**Figure 8:**
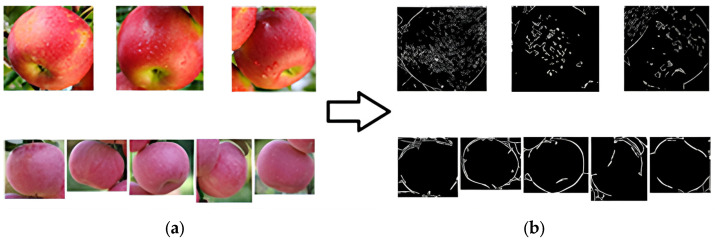
Effect of edge detection without clustering. (**a**) shows the raw images extracted from the object detection module without further processing. (**b**) shows the result obtained by directly applying edge detection to (**a**), displaying issues such as speckle noise, discontinuous edges, and lack of clarity.

After being cut by the Faster-RCNN model, the image still contained irrelevant features such as leaves and branches, resulting in noise and voids when directly performing edge detection (as shown in Figure 8). For this problem, the K-means clustering method was used to cluster and segment the image to be processed, extract the target part of the image, and remove irrelevant features around the target. By conducting experiments, it was determined that setting the value of k for K-means clustering to 2 resulted in the best clustering performance.

The original images were all in RGB format, as shown in Figure 8a. The RGB color space is based on three basic colors, R (red: red), G (green: green), and B (blue: blue), without separating brightness information from chromaticity information. Clustering and segmenting images in RGB color space can result in a significant loss of target edge pixel information, leading to a significant gap between the edge detection effect of the target and the actual effect as shown in Figure 8b.

It was necessary to convert the image from the RGB space to the LAB space to minimize the pixel information loss of the target. The LAB space effectively separated brightness information and chromaticity information, where L represents brightness and a and b represent color channels.

**Figure 9:**
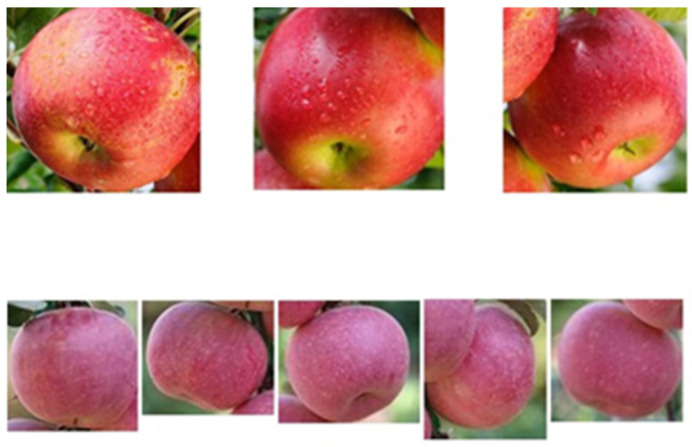
Edge detection model input image examples.

**Figure 10:**
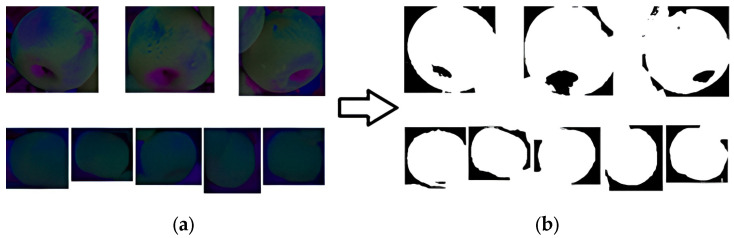
K-means clustering for image segmentation. (**a**) shows the effect of converting single apple images into a Lab color space. (**b**) shows the results after applying K-means clustering to (**a**). The upper part of (**b**) contains voids due to the depression at the bottom of the fruit, while the lower part (**b**) only contains a few small voids, resulting in a relatively complete overall clustering effect.

The input image examples of the edge detection model are shown in Figure 9. The spatial transformation results for K-means clustering and cutting are shown in Figure 10.

#### 2.5.2. Image Denoising

**Figure 11:**
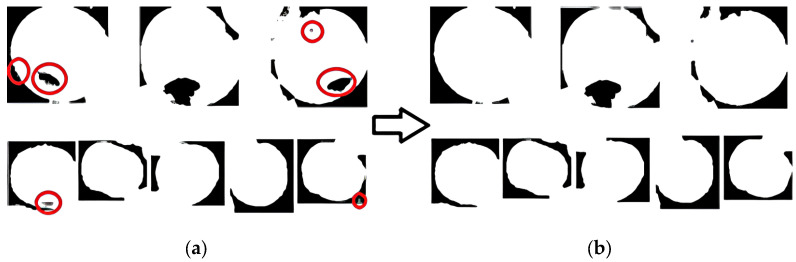
Example diagram image processing effect after clustering and segmentation. (**a**) shows the effect of labeling defects such as voids after clustering. The red circle denotes the hole that exists after the segmentation. (**b**) shows the effect of morphological processing such as dilation, erosion, and filling in voids on the images, effectively removing most of the existing defects.

The clustering results in Figure 10 show that there were voids and edge noise effects in the image. This study adopted the void-filling algorithm to address the problem of voids. For edge noise, the erosion method was used to process the image edges, but simple erosion processing lost the pixel information of the target edge. Therefore, the dilation method was used to process the corroded image. The results are shown in Figure 11.

The images were filled with voids, eroded, and dilated, although after processing in this stage, the voids in the image were filled, edge noise was eliminated, and there was basically no loss of pixel information at the apple edge.

### 2.6. Edge Detection Module

The rough set method was the core method of this study. Currently, there are many traditional edge detection operators, such as Canny [32,33,34], Laplacian [35,36], Prewitt [37,38], and Holistically-Nested(HED) [39,40,41]. Many research papers have shown that traditional operators also have better edge detection effects.

In this paper, a structural operator for upper and lower approximation operations was defined based on rough set theory. The process of traversing objects using the structural operator convolution method to obtain the upper approximation of the image is as follows.

(1) Assume that the target object image X, the RS structure operator Y is defined, and the initial U(X) is empty.
(2) First, the structural operator Y is placed in the region corresponding to the size in the upper left corner of X, and the elements X [i, j] in object X are overlapped with those in Y. The X [i, j] and Y [i, j] bitwise AND operations are computed. If the detection point is inside the object, it is considered to belong to U (X), and there is a high possibility of points belonging to the object around it. U (X) is added, and the sliding structural operator Y continues until the traversal is completed and the process stops. Finally, the maximum pixel value of the object coverage area can be obtained.
(3) The U (X) obtained after traversal is the upper approximation of the edge of the target object.

The process of traversing objects using the structural operator convolution method to obtain the lower approximation of the image is as follows.

(1) Assuming the target object image X, define the rough set structure operator Y, and the initial U (X) is empty.
(2) The structural operator is first placed in the area corresponding to the size in the upper left corner, and the elements [i, j] in the objects in that area are aligned with the elements in Y. Compute [i, j] and [i, j] bitwise OR operations. If the detection point is inside the object, it is considered to belong to $L(X^{\prime })$. If the detection point belongs to an edge point, then there are points with different pixel values around it. There is a high possibility of edge points around it, so they are added to L(X′). The structural operator $Y^{\prime }$ is continuously slid until $X^{\prime }$ has been traversed before stopping. Finally, the minimum pixel value of the object coverage area is obtained.
(3) $L(X^{\prime })$ obtained after traversal is the lower approximation of the edge of the target object.

**Figure 12:**
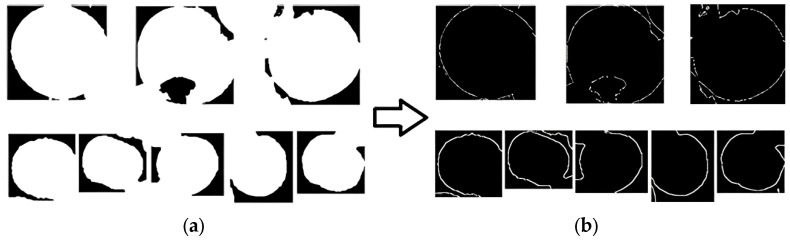
Obtaining apple edge saliency profile images using rough set. (**a**) shows the resulting images after morphological processing. (**b**) shows the effect of edge detection based on the rough set method.

In this study, rough set was used to obtain the upper and lower approximation edges of the target. Referring to the method for computing Bn(X) as per Formula (3), we have successfully obtained the salient contours of the target. The edge detection effect is shown in Figure 12.

### 2.7. Edge Consolidation

**Figure 13:**
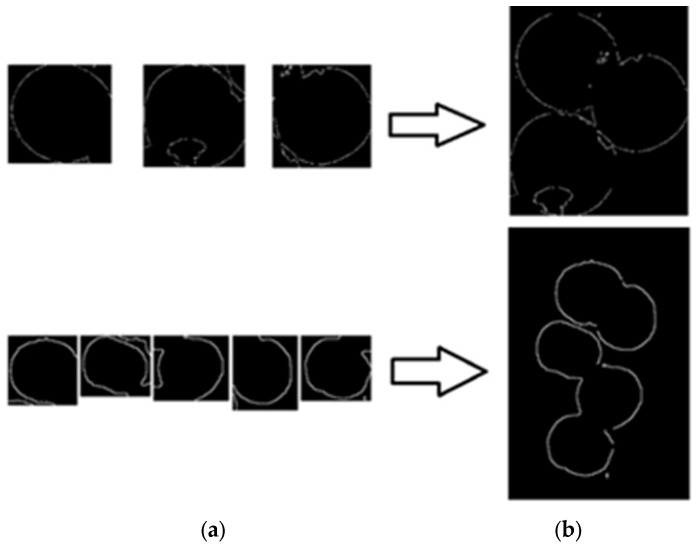
Effect of apple edge image merge. (**a**) shows the result images after edge detection. (**b**) shows the merging of all edge images into the corresponding edge image of the original image based on the recorded coordinates of each apple image.

Considering the excessive noise caused by multiple apples, an object detection algorithm based on the Faster-RCNN was adopted to segment the images. The segmented multiple images were input into the edge detection model to obtain multiple single apple edge result images, as shown in Figure 13a. The position information of each segmented image obtained via the object detection algorithm in the original image was used to draw multiple edge images of the same size to restore multiple edge images to a multi-apple image, as shown in Figure 13b.

### 2.8. Evaluation Metrics

The results of this paper were evaluated via four indicators: precision (P), recall rate (R), Dice (D), and Jaccard (J).

Accuracy was an important indicator in the performance evaluation of classifiers and was used to measure the accuracy of classifiers for samples with positive predictions. It can be expressed as the following formula:

(4) $$Precision=\frac{TP}{TP+FP}$$

where TP represents the number of samples correctly predicted as positive examples, and FP represents the number of samples incorrectly predicted as positive examples.

The recall rate was another core classifier performance evaluation indicator and was used to measure the recall rate of the classifier for actual positive samples. It can be expressed as the following formula:

(5) $$Recall=\frac{TP}{TP+FN}$$

where TP represents the number of samples correctly predicted as positive examples, and FN represents the number of samples incorrectly predicted as negative examples.

The Dice coefficient (Sørensen-Dice Dice coefficient) and Jaccard index are two indicators for measuring similarity, and the calculation formulas are as follows [42]:

(6) $$Dice=\frac{2\times TP}{2\times TP+FP+FN}$$

(7) $$Jaccard=\frac{TP}{TP+FP+FN}$$

where TP represents the number of samples correctly predicted as positive examples, and FP represents the number of samples incorrectly predicted as positive examples, and FN represents the number of samples incorrectly predicted as negative examples.

The aforementioned metrics were all calculated using a confusion matrix and are commonly used for object detection tasks. We constructed the confusion matrix for edge detection results and actual edge results using the following method.

We employed two N × N filters (in the experiment, we used 3 × 3, the filter size can be adjusted reasonably based on the actual error tolerance) to slide simultaneously over the result image (filter B) and the ground truth edge image (filter A). There were four possible scenarios:

True Positive (TP): both filters detect an edge pixel.

False Positive (FP): only Filter B detects an edge pixel, but Filter A does not.

False Negative (FN): only Filter A detects an edge pixel, but Filter B does not.

True Negative (TN): neither filter detects an edge pixel.

Additionally, we evaluated the segmentation results of the images involved using two metrics: mask_mAP and area relative error. The calculation method for area relative error is as follows:

(8) $$\Delta E_{s}=\left|S_{1}-S_{2}\right|$$

(9) $$r_{s}=\frac{\Delta E_{s}}{S_{1}}$$

where, S1 is the number of pixels in the actual apple, S2 is the number of pixels in the apple contour.

## 3. Results and Discussion

In this study, comparative experiments were conducted using Canny, Laplacian, Pewitt, and Holistically-Nested object detection operators and the rough set edge detection algorithm based on the object detection proposed in this paper. Comparative analyses were conducted for three aspects: illumination influence, complex background influence, and dense occlusions influence.

### 3.1. Analysis of Detection Results Using the Segment Anything Model (SAM)

The segment anything model (SAM) aims to segment objects of interest in images based on user-provided cues. Its strength lies in having learned the concept of objects, enabling it to segment any object. Since our work involves object segmentation, it was interesting and necessary to use SAM for the segmentation of our data in the experimental section and to discuss the results.

**Figure 14:**
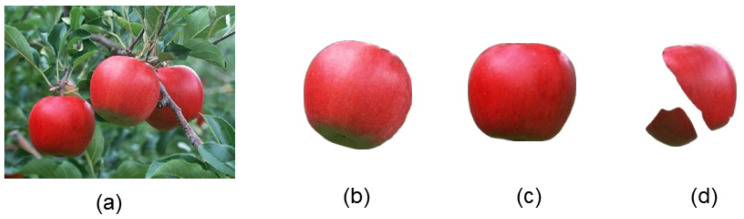
The original image and the segmentation results of the sparse fruit image using SAM. (**a**) represents the original image, while (**b**–**d**) display the results after SAM segmentation.

We conducted experiments with different apple fruit images captured in natural environments using SAM. Figure 14a is a simple example image containing only three easily recognizable apples. The original image and the segmentation results are shown in Figure 14.

As shown in Figure 14b,c, we can observe that the segmentation results for unobstructed objects were nearly perfect, with minimal edge loss. However, for objects that were occluded, such as occlusion between fruits or obscured by branches, as shown in Figure 14d, the segmentation results were relatively poor, with obvious missing parts in the occluded areas.

**Figure 15:**
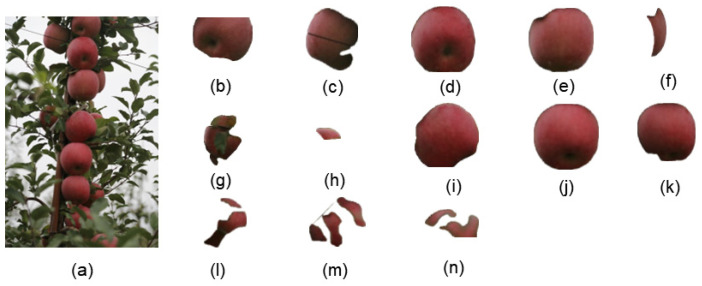
The original image and the segmentation results of the dense fruit image using SAM. (**a**) represents the original image, while (**b**–**n**) display the results after SAM segmentation.

Figure 15a is a complex image example with densely packed apples and numerous occlusion factors. The original image and segmentation results are shown in Figure 15.

From Figure 15, it can be observed that Figure 15d,e,j are relatively complete, although there was some loss of edge pixels. However, other objects experienced varying degrees of pixel loss due to occlusion by leaves or mutual occlusion between fruits, especially in segments Figure 15f–h,l–n.

Overall, SAM can achieve remarkable segmentation results without the need for specific scene data training. However, because SAM requires achieving high-quality segmentation for every object, it cannot focus on preserving complete apples in the groundwork for edge detection. In other words, the underlying concept behind SAM is that branches, leaves, and fruits were all segmentation targets. Because SAM achieves precise segmentation for each part, there was a loss of fruit obscured by branches and leaves, which deviated from our goal of preserving the apples as completely as possible.

### 3.2. Analysis of the Detection Results on the Effect of Illumination

**Figure 16:**
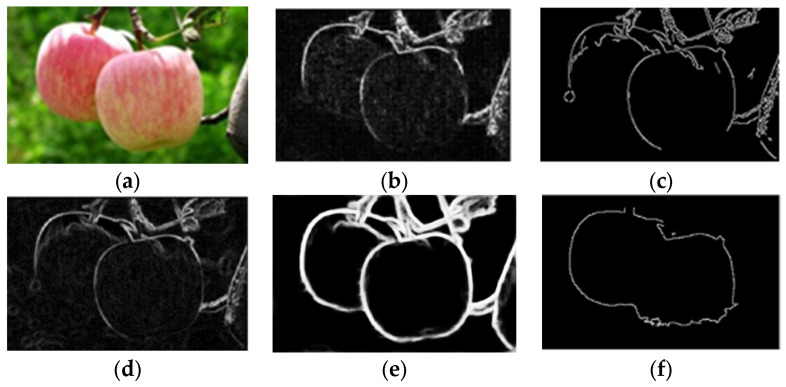
Comparison of the effects of each method under the influence of illumination. (**a**) shows the original image before detection; (**b**) shows the apple edges detected by the Canny operator; (**c**) shows the apple edges detected by the Laplacian operator; (**d**) shows the apple edges detected by the Prewitt operator; (**e**) shows the apple edges detected via the Holistically-Nested operator; and (**f**) shows the apple edges detected via the model in this study.

To compare the detection results of different algorithms under different illumination conditions, the Canny, Laplacian, Prewitt, and Holistically-Nested operators and the method proposed in this paper were used for detection experiments; the results are shown in Figure 16.

Figure 16a shows the original image for detection, and the upper end of the apple in the legend has a reflective problem due to the influence of illumination. The results in Figure 16 show that the edges detected via the Laplacian and Prewitt operators retained more noise, and the edge shape was thicker. The edge continuity of the apple image detected via the Canny operator was not strong, and the edge information of the illumination part was lost, which was greatly affected by noise. The edge detection results obtained via the Holistically-Nested method surpassed those of the previous operators; however, it still faced challenges in handling interferences such as branches and leaves. Under the influence of illumination, the edge detection model that we improved resulted in relatively complete edge information detected within the apple image. Compared with the Canny operator, Laplacian operator, Prewitt operator, and Holistically-Nested operator methods in the comparative experiments, it can be observed from Figure 16 that the apple edge detection information obtained using our method performed better in terms of completeness and conciseness. However, there exists a problem of discontinuous edges due to occlusion leading to covered edge locations.

### 3.3. Analysis of Detection Results on the Effect of Complex Background

**Figure 17:**
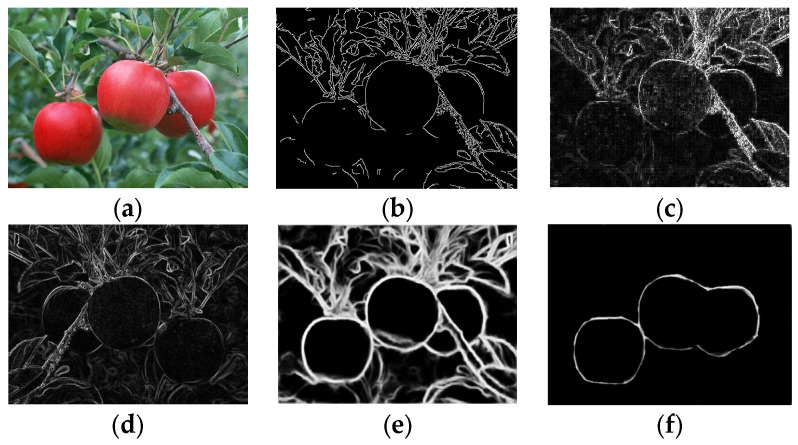
Comparison of the effects of each method under the influence of a complex background. (**a**) shows the original image before detection; (**b**) shows the apple edges detected via the Canny operator; (**c**) shows the apple edges detected via the Laplacian operator; (**d**) shows the apple edges detected via the Prewitt operator; (**e**) shows the apple edges detected via the Holistically-Nested operator; and (**f**) shows the apple edges detected via the algorithm in this paper.

Considering the noise caused by the complex background of the apple in actual environmental images, edge detection experiments were conducted using the above algorithm sequentially on the apple in a complex background. The results are shown in Figure 17.

The results in Figure 17 show that operators such as Canny, Laplacian, and Prewitt did not eliminate edge information from backgrounds such as leaves and branches. The Holistically-Nested method provided a clear depiction of the edges of fruits, but it similarly did not remove the influence of branches or leaves, as shown in Figure 17e.

Using our method, as shown in Figure 17f, we effectively removed irrelevant objects such as branches and leaves. In the detection results, there was no point-like noise interference within the fruit. It is worth noting that the fruits on the right did not exhibit edge loss or discontinuity due to branch occlusion. This was attributed to the previous morphological processing of images and rough set, which mitigated interference and filled in defects.

### 3.4. Analysis of Results on the Effect of Dense Occlusions

**Figure 18:**
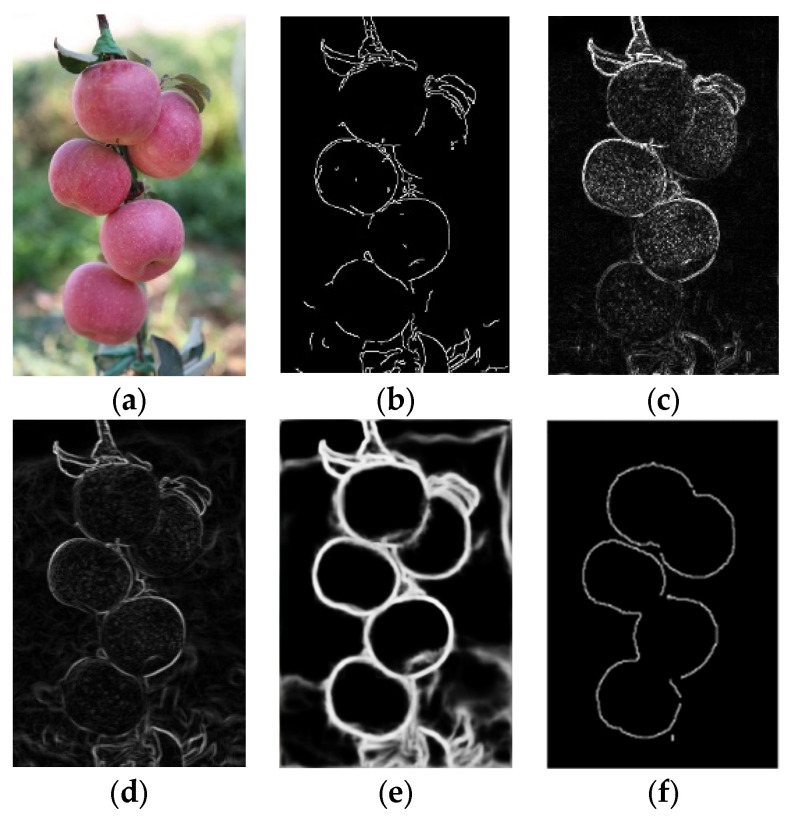
Comparison of the effects of the algorithms under the influence of dense occlusions. (**a**) shows the original image before detection. There were multiple closely connected apples in the legend, and the apples interacted with each other. (**b**) shows the apple edges detected via the Canny operator. (**c**) shows the apple edges detected via the Laplacian operator. (**d**) shows the apple edges detected via the Prewitt operator. (**e**) shows the apple edges detected via the Holistically-Nested operator. (**f**) shows the apple edges detected via the algorithm in this paper.

For the situation where apples were densely distributed in the images, comparative detection experiments were conducted based on the above algorithms, and the results are shown in Figure 18.

According to Figure 18b, under a dense apple distribution, the Canny operator cannot effectively distinguish noise and detect targets, resulting in excessive noise and discontinuous edges being detected due to the dense apple distribution. Figure 18c shows that the Laplacian operator was highly coherent for edge detection between dense apples, but it did not have good noise resistance and was too sensitive to spots on apples, resulting in considerable point-like noise inside the target. Figure 18d shows that the Prewitt operator did not have good noise resistance and that there were many points, such as noise inside the target. Figure 18d indicates that the Prewitt operator did not have good noise resistance and that there was a lot of point noise inside the target. Figure 18e shows the detection results obtained using the Holistically-Nested method, which outperformed previous operators in terms of the clarity of edge delineation and the degree of internal speckle noise removal. However, it tended to capture extraneous edges influenced by tree branches and leaves. Figure 18f shows that our proposed model had significant noise resistance and had a significant effect on edge detection of dense apples. It also effectively eliminated speckle noise inside apples. However, the issue of discontinuous coverage in the detected regions due to fruit occlusion is also an aspect that requires improvement.

**Figure 19:**
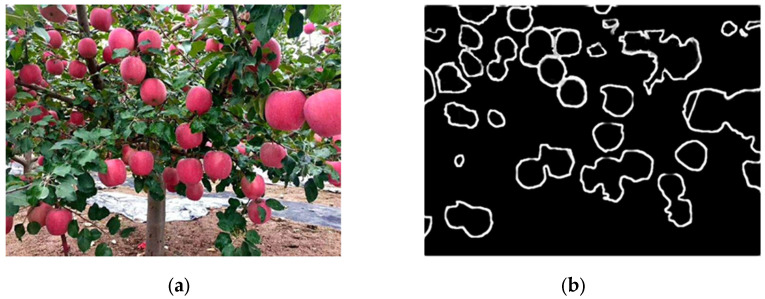
The edge detection experiments on images with more complex content. (**a**) shows the original image before detection. (**b**) shows the processed edge detection results.

We further conducted experiments on images with more complex content, and the results are shown in Figure 19.

Figure 19a shows the original image for detection, which contains a significant number of apple targets. The increased content resulted in lower resolution of the apple targets and more complex depth relationships among them. From Figure 19b, it can be observed that the targets located at the surface of the image are depicted with relatively clear edges. The edge detection results for the apple targets located deeper within the image exhibited varying degrees of incompleteness. Overall, the presence of multiple layers of occlusion and resolution issues both affect the effectiveness of edge detection. In real complex large-scale scenes, the performance of this method for edge detection was not ideal.

### 3.5. Ablation Study

#### 3.5.1. Validation of the Effectiveness of the Key Modules

We carried out an ablation experiment to verify the effectiveness of each key module. The purpose of the object detection module was to locate and identify objects of interest in the image.

Table 1 shows that when this module worked independently, background noise, illumination changes, or other factors may have interfered. We further refined the task when we introduced the clustering segmentation module so that the system could better distinguish between target and nontarget regions. The purpose of clustering was to group pixels or features based on certain similarity indicators, such as color, texture, or shape, to provide clearer target boundaries. Finally, the morphological processing module provided a way to process images to further enhance or refine the shape and structure of the target. The introduction of morphological operations such as corrosion and expansion significantly improved the elimination of noise, filled in blank areas in the target, and highlighted the main features of the target. From Table 1, it can be concluded that both the recall rate and F1-score increased by 7.3%, and the precision value increased by 7.2% after introducing the cluster segmentation module. After introducing the erosion and dilation module, the precision value increased by 7.7%, the recall rate increased by 4.0%, and the F1-score increased by 5.7%.

#### 3.5.2. Clustering Segmentation K-Value Experiment

Table 2 shows that too many categories can lead to segmenting fruit regions, resulting in errors. However, if there are too few cluster centers, the fruits cannot be completely separated from the background. This was because the elements in the image were roughly divided into three categories: fruit, background, and noise. In our experiment, the algorithm achieved the best performance when the number of cluster centers was fixed at 3 as it achieved 88.3% accuracy, 93.6% recall, and 90.9% F1-score.

#### 3.5.3. Erosion and Dilation Experiments

To fully verify the influence of the number of iterations of erosion and dilation, we further compared the models with different numbers of iterations. Table 3 shows that the F1-score will decrease when the number of iterations is too large or too small. In particular, having too many control points will seriously degrade the performance. When the number of control iterations is too large, the integrity of the image will greatly decrease. The best performance was achieved when the number of iterations was approximately three. Therefore, in our experiment, the number of iterations for corrosion expansion was fixed at three.

## 4. Overall Analysis of the Experimental Results

**Figure 20:**
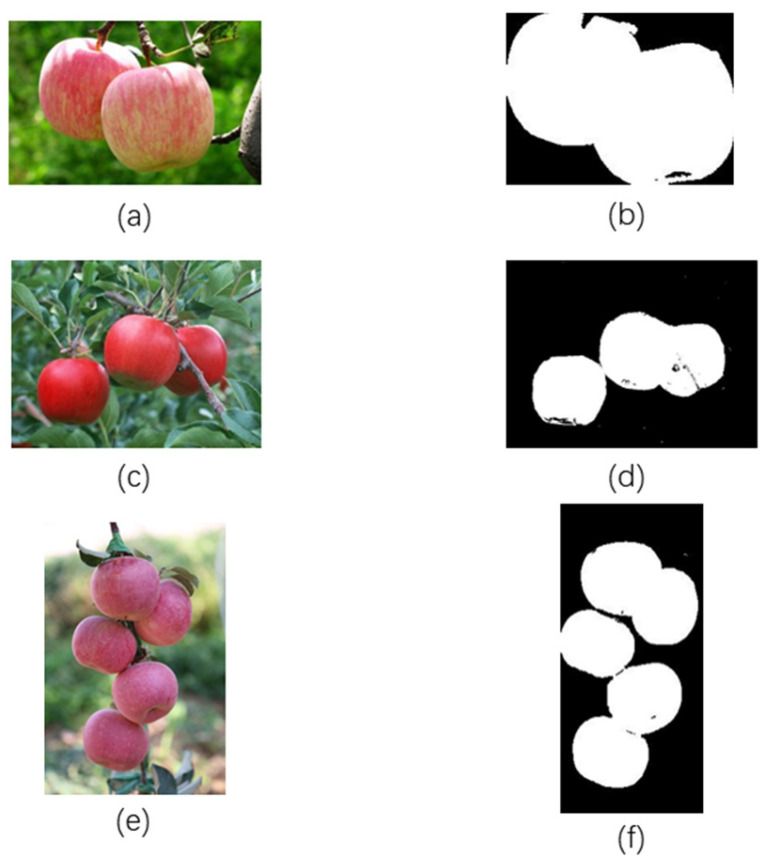
Illustration of the segmentation module. (**a**) shows the original image affected by illumination before segmentation. (**b**) shows the image affected by illumination after segmentation using our method. (**c**) shows the original image affected by complex backgrounds before segmentation. (**d**) shows the image affected by complex backgrounds after segmentation using our method. (**e**) shows the original image affected by dense occlusions before segmentation. (**f**) shows the image affected by dense occlusions after segmentation using our method.

Using our method, we conducted experiments in three scenarios: illumination, complex backgrounds, and dense occlusions. In the illumination scenario, the images contained the least number of apples, while in the dense occlusions scenario, the images contained the highest number of apples. An illustration of the segmentation module is shown in Figure 20.

We evaluated the image segmentation performance in the three experimental scenarios, as shown in Table 4. From Table 4, it can be observed that as the number of targets increases, the Mask_mAP value gradually decreases. This indicated that with the increase in the number of apples, the segmentation performance was affected to some extent, but remained within a relatively ideal range.

The area relative error values exhibited a small range of variation and small numerical values, indicating that regardless of changes in influencing factors or an increase in the number of apples, the final edge detection results were essentially unaffected and remained close to the original position.

Common deep learning segmentation methods such as U-Net [43], SegNet [44], and Mask-RCNN [45] require the creation of training data for model training. Our segmentation method exclusively utilizes K-Means for pixel-level segmentation, representing an unsupervised approach. This method provides a feasible solution for effectively describing apples in situations where annotated data is scarce. This is particularly important in practical applications because in many real-world scenarios, acquiring a large amount of accurately annotated data is both time-consuming and costly. Of course, for more complex scenarios and when abundant accurately annotated data is available, choosing deep learning-based methods is more effective.

We compared the edge detection results of the proposed model with those of the Canny operator, Laplacian operator, Prewitt operator, and Holistically-Nested operator in terms of three aspects: illumination effect, complex background effect, and dense occlusion effect, as shown in Table 5.

Table 5 shows that the model proposed in this paper exhibited better adaptability in various aspects, such as the effects of illumination, complex backgrounds, and dense occlusions. In terms of illumination effect experiments, the precision value, recall rate, dice, and Jaccard of our algorithm were 82.6%, 94.4%, 88.1%, and 78.7%, except that the precision value was slightly lower than the Holistically-Nested operator. Other evaluation indexes were better than the Canny operator, Laplacian operator, Prewitt operator, and Holistically-Nested operator of the contrast experiments. In terms of complex background effect experiments, the precision value, recall rate, dice, and Jaccard of our algorithm were 88.3%, 93.6%, 90.9%, and 83.3%, and the detection effect was superior to the Canny operator, Laplacian operator, Prewitt operator, and Holistically-Nested operator. In terms of dense occlusions effect experiments, the precision value, recall rate, dice, and Jaccard of our algorithm were 83.8%, 97.6%, 90.2%, and 82.1%, except that the precision value was slightly lower than Canny operator and Holistically-Nested operator. The detection effect was superior to the Canny operator, Laplacian operator, Prewitt operator, and Holistically-Nested operator.

The analysis of the experimental results revealed that the model in this paper achieved noise reduction by gradually removing the environmental noise around the target fruit. Our proposed rough set edge detection method can better utilize pixel information with fuzzy fruit edges and unclear classification, more effectively extract the salient contours of fruits, and overall demonstrate stronger robustness.

## 5. Conclusions

Based on the application background of target edge detection, a detection method combining rough set and convolutional neural network was proposed in this paper. This method obtains the target edge image by gradually extracting the target features of the original image and minimizing the loss of target features through graphics-related methods.

In response to the problems of multiple apples in the original image, which contain too many irrelevant features and severe mutual influence between apples, a Faster-RCNN model was constructed using convolutional neural network knowledge in deep learning to segment multiple apples one by one and simplify the multi-apple edge detection problem into a single apple edge detection problem.

For edge detection of a single segmented apple image, the branches and leaves around the target still have a serious impact. According to various related studies, the K-means clustering method was used to segment the target from the background and achieved further noise reduction. In the processed results, the target image still suffered from feature loss and a small amount of edge noise. To address this, we used cavity filling and graphic erosion and dilation methods to minimize the loss of target features while maximizing noise removal.

In terms of the core algorithm of edge detection, the rough set method was introduced to better characterize the edge image of the target in response to the uncertainty and imprecision of image edges. The experimental results showed that, considering the effects of illumination, complex backgrounds, and dense occlusions, the values of dice were 88.1%, 90.9%, and 90.2%, respectively, which were significantly higher than those of the Canny operator, Laplacian operator, Prewitt operator, and Holistically-Nested operator participating in the contrast experiment. Meanwhile, the values of Jaccard were 78.7%, 83.3%, and 82.1%, respectively, which were higher than those of the Canny operator, Laplacian operator, Prewitt operator, and Holistically-Nested operator participating in the contrast experiment.

This paper studied the positioning and edge detection of apples. The research objects of this paper were apple target location and edge detection in the natural environment, and the accuracy of apple detection was effectively improved, which provides a valuable reference for intelligent harvesting, growth analysis, and yield prediction. However, there are still some areas that require improvement. We have designed a series of processes to overcome issues such as branch occlusion, fruit occlusion, and shadow interference. Although these processes largely remove non-target pixels, the final edge detection results revealed that occlusion between fruits can lead to discontinuous edge delineation. Continuous edge delineation is crucial for capturing depth information between targets. In future work, we need to improve the issue of discontinuous edges, such as by introducing depth information to fill in the final discontinuous edges.

## Figures and Tables

**Table 1:** Module ablation experiment. The ✓ indicates that this module was added to the ablation experiment.

Object Detection Module	Cluster Segmentation Module	Erosion and Dilation Module	P/%	R/%	F/%
✓			73.4	82.3	77.6
✓	✓		80.6	89.6	84.9
✓	✓	✓	88.3	93.6	90.8

**Table 2:** Comparison data of cluster values of clustering modules.

K	P/%	R/%	F/%
2	81.3	83.6	82.4
3	88.3	93.6	90.9
4	79.2	86.5	82.7

**Table 3:** Comparison data of erosion and dilation iterations.

Iterations	P/%	R/%	F/%
1	86.2	91.4	88.7
2	87.9	92.3	90
3	88.3	93.6	90.9
5	78.1	82.8	80.4
10	63.1	66.2	64.6

**Table 4:** Image segmentation metrics under different scenarios.

Condition	Mask_mAP/%	Area Relative Error/%
Illumination	94.1	4.67
Complex Backgrounds	92.7	5.99
Dense Occlusions	90.6	6.14

**Table 5:** Comparison data of various methods.

Algorithm	Illumination Effect				Complex Backgrounds Effect				Dense Occlusions Effect			
	P/%	R/%	D/%	J/%	P/%	R/%	D/%	J/%	P/%	R/%	D/%	J/%
Canny	84.6	87.2	85.8	75.3	63.7	92.6	75.5	60.6	87.9	79.9	83.7	72.0
Laplacian	74.3	86.2	79.7	66.4	40.1	88.5	55.2	38.1	82.2	95.1	88.1	78.8
Prewitt	80.1	90.8	85.1	74.1	78.8	88.3	83.2	71.3	72.5	90.4	80.4	67.3
Hed	84.8	91.1	87.8	78.3	87.7	92.4	89.9	81.8	86.3	90.5	88.3	79.1
Ours	82.6	94.4	88.1	78.7	88.3	93.6	90.9	83.3	83.8	97.6	90.2	82.1

## Data Availability

Data are available on request.

## References

[1] Chen R., Wang J., Li Y., Song Y., Huang M., Feng P., Qu Z., Liu L. (2023). Quantifying the impact of frost damage during flowering on apple yield in Shaanxi province, China. Eur. J. Agron..

[2] Versaci M., Morabito F.C. (2021). Image Edge Detection: A New Approach Based on Fuzzy Entropy and Fuzzy Divergence. Int. J. Fuzzy Syst..

[3] Joo J., Han L., Tian Y., Qi Q. (2020). Research on edge detection algorithm based on improved sobel operator. MATEC Web Conf..

[4] Lu Y., Duanmu L., Zhai Z., Wang Z. (2022). Application and improvement of Canny edge-detection algorithm for exterior wall hollowing detection using infrared thermal images. Energy Build..

[5] Akbari Sekehravani E., Babulak E., Masoodi M. (2020). Implementing canny edge detection algorithm for noisy image. Bull. Electr. Eng. Inform..

[6] Septiarini A., Hamdani H., Hatta H.R., Anwar K.J.S.H. (2020). Automatic image segmentation of oil palm fruits by applying the contour-based approach. Sci. Hortic..

[7] Jiao Y., Luo R., Li Q., Deng X., Yin X., Ruan C., Jia W. (2020). Detection and Localization of Overlapped Fruits Application in an Apple Harvesting Robot. Electronics.

[8] Su Z., Liu W., Yu Z., Hu D., Liao Q., Tian Q., Pietikäinen M., Liu L. Pixel difference networks for efficient edge detection. Proceedings of the IEEE/CVF International Conference on Computer Vision.

[9] Wang D., He D., Song H., Liu C., Xiong H. (2019). Combining SUN-based visual attention model and saliency contour detection algorithm for apple image segmentation. Multimed. Tools Appl..

[10] Ganesan P., Sathish B., Sajiv G. Automatic segmentation of fruits in CIELuv color space image using hill climbing optimization and fuzzy C-Means clustering. Proceedings of the 2016 World Conference on Futuristic Trends in Research and Innovation for Social Welfare (Startup Conclave).

[11] Poma X.S., Riba E., Sappa A. Dense extreme inception network: Towards a robust cnn model for edge detection. Proceedings of the IEEE/CVF Winter Conference on Applications of Computer Vision.

[12] Wang D., He D. (2022). Apple Detection and Instance Segmentation in Natural Environments Using an Improved Mask Scoring R-CNN Model. Front. Plant Sci..

[13] Tian Y., Yang G., Wang Z., Wang H., Li E., Liang Z. (2019). Apple Detection during Different Growth Stages in Orchards Using the Improved YOLO-V3 Model. Comput. Electron. Agric..

[14] Li Q., Jia W., Sun M., Hou S., Zheng Y. (2021). A Novel Green Apple Segmentation Algorithm Based on Ensemble U-Net under Complex Orchard Environment. Comput. Electron. Agric..

[15] Zhang C., Zou K., Pan Y. (2020). A Method of Apple Image Segmentation Based on Color-Texture Fusion Feature and Machine Learning. Agronomy.

[16] Ren S., He K., Girshick R., Sun J. Faster r-cnn: Towards real-time object detection with region proposal networks. Proceedings of the Advances in Neural Information Processing Systems 28 (NIPS 2015).

[17] Bao J., Wei S., Lv J., Zhang W. Optimized faster-RCNN in real-time facial expression classification. Proceedings of the IOP Conference Series: Materials Science and Engineering.

[18] Yu X., Yuan Y.J.J.C. (2019). Hand Gesture Recognition Based on Faster-RCNN Deep Learning. J. Comput..

[19] Zhang K., Shen H.J.A.S. (2021). Solder joint defect detection in the connectors using improved faster-rcnn algorithm. Appl. Sci..

[20] Teng B., Zhao H., Jia P., Yuan J., Tian C. (2020). Research on ceramic sanitary ware defect detection method based on improved VGG network. J. Phys..

[21] Sarwinda D., Paradisa R.H., Bustamam A., Anggia P. (2021). Deep learning in image classification using residual network (ResNet) variants for detection of colorectal cancer. Procedia Comput. Sci..

[22] Guo Y., Zhang J., Su P., Hou G.H., Deng F.Y. (2020). The Study of Locating Diseased Leaves Based on RPN in Complex Environment. J. Phys..

[23] Retter T.L., Webster M.A.J.C.B. (2021). Color Vision: Decoding Color Space. Curr. Biol..

[24] Pardede J., Husada M.G., Hermana A.N., Rumapea S.A. Fruit ripeness based on RGB, HSV, HSL, L ab color feature using SVM. Proceedings of the 2019 International Conference of Computer Science and Information Technology (ICoSNIKOM).

[25] Khan I., Luo Z., Shaikh A.K., Hedjam R. (2021). Ensemble clustering using extended fuzzy k-means for cancer data analysis. Expert Syst. Appl..

[26] Sharma P., Sharma M.S.P., Tomar R.S., Sciences O. (2017). A new approach for image segmentation using improved k-means and ROI saliency map. J. Inf. Optim. Sci..

[27] Yuan Y., Shi B., Yost R., Liu X., Tian Y., Zhu Y., Cao W., Cao Q.J.P. (2022). Optimization of Management Zone Delineation for Precision Crop Management in an Intensive Farming System. Plants.

[28] An S., Hu Q., Wang C. (2021). Probability granular distance-based fuzzy rough set model. Appl. Soft Comput..

[29] Atef M., Nada S.I. (2021). On three types of soft fuzzy coverings based rough sets. Math. Comput. Simul..

[30] Raj A., Minz S. (2021). A Scalable Unsupervised Classification Method Using Rough Set for Remote Sensing Imagery. Int. J. Softw. Sci. Comput. Intell..

[31] Khanzadi P., Majidi B., Adabi S., Patra J.C., Movaghar A. (2021). Robust fuzzy rough set based dimensionality reduction for big multimedia data hashing and unsupervised generative learning. Multimed. Tools Appl..

[32] Nnolim U.A. (2020). Automated crack segmentation via saturation channel thresholding, area classification and fusion of modified level set segmentation with Canny edge detection. Heliyon.

[33] Yang Y., Zhao X., Huang M., Wang X., Zhu Q.J.C. (2021). Multispectral image based germination detection of potato by using supervised multiple threshold segmentation model and Canny edge detector. Comput. Electron. Agric..

[34] Zhang H., Tang K., Zhang C., Pan S., Yang Z. (2021). Image segmentation based on GGVF Snake model and Canny operator. Sci. J. Intell. Syst. Res..

[35] Dinkar S.K., Deep K., Mirjalili S., Thapliyal S. (2021). Opposition-based Laplacian equilibrium optimizer with application in image segmentation using multilevel thresholding. Expert Syst. Appl..

[36] Lorencin I., Anđelić N., Španjol J., Car Z. (2020). Using multi-layer perceptron with Laplacian edge detector for bladder cancer diagnosis. Artif. Intell. Med..

[37] Krishnan Nair S., Chinnappan S.K., Dubey A.K., Subburaj A., Subramaniam S., Balasubramaniam V., Sengan S. (2023). Engineering. Prewitt Logistic Deep Recurrent Neural Learning for Face Log Detection by Extracting Features from Images. Arab. J. Sci. Eng..

[38] Zhou R.-G., Yu H., Cheng Y., Li F.-X. (2019). Quantum image edge extraction based on improved Prewitt operator. Quantum Inf. Process..

[39] Xu W., Thomasson J.A., Su Q., Ji C., Shi Y., Zhou J., Chen H. (2022). A segmentation algorithm incorporating superpixel block and holistically nested edge for sugarcane aphids images under natural light conditions. Biosyst. Eng..

[40] Xu S., Hao M., Liu G., Meng Y., Han J., Shi Y. (2022). Concrete crack segmentation based on convolution–deconvolution feature fusion with holistically nested networks. Struct. Control Health Monit..

[41] Cheng R., Alexandridi N.A., Smith R.M., Shen A., Gandler W., McCreedy E., McAuliffe M.J., Sheehan F.T. (2020). Fully automated patellofemoral MRI segmentation using holistically nested networks: Implications for evaluating patellofemoral osteoarthritis, pain, injury, pathology, and adolescent development. Magn. Reason. Med..

[42] Bolboacă S.D., Jäntschi L. (2014). Sensitivity, specificity, and accuracy of predictive models on phenols toxicity. J. Comput. Sci..

[43] Ronneberger O., Fischer P., Brox T., Navab N., Hornegger J., Wells W.M., Frangi A.F. (2015). U-Net: Convolutional Networks for Biomedical Image Segmentation. Medical Image Computing and Computer-Assisted Intervention—MICCAI.

[44] Badrinarayanan V., Kendall A., Cipolla R. (2017). SegNet: A Deep Convolutional Encoder-Decoder Architecture for Image Segmentation. IEEE Trans. Pattern Anal. Mach. Intell..

[45] Yu Y., Zhang K., Yang L., Zhang D. (2019). Fruit Detection for Strawberry Harvesting Robot in Non-Structural Environment Based on Mask-RCNN. Comput. Electron. Agric..

